# Translation and Psychometric Evaluation of the Arabic Version of the Eating Disorders After Bariatric Surgery Questionnaire (EDABS‑Q‑Arabic18)

**DOI:** 10.1007/s11695-025-07910-9

**Published:** 2025-06-17

**Authors:** Mohamed Hany, Kareem El-Ansari, Walid El Ansari

**Affiliations:** 1 Alexandria University, Alexandria, Egypt; 2 Madina Women’s Hospital, Alexandria University, Alexandria, Egypt; 3 St. George’s University, St. George’s, Grenada; 4 Ajman University, Ajman, United Arab Emirates

**Keywords:** Metabolic and bariatric surgery, Disordered-eating behaviors, Eating disorders, Eating pathology, Questionnaire, Arabic translation, Validation

## Abstract

**Background:**

There is no validated Arabic questionnaire to assess eating disorders (ED) after metabolic and bariatric surgery (MBS) without the need for an interview. We undertook this task.

**Methods:**

The English-published Eating Disorder Examination-Self-report Questionnaire Bariatric Surgery Version (EDABS-Q) was translated and adapted following international guidelines (forward/backward translation, expert panel review, and pretesting). Randomly selected adult patients (*n* = 1145) who had MBS since ≥ 1 year completed the Arabic questionnaire (EDABS-Q-Arabic). Psychometric properties of EDABS-Q-Arabic were assessed, including face validity (expert panel), construct validity (exploratory factor analysis, confirmatory factor analysis, structural model), internal consistency (Cronbach’s *α*), and discriminant validity [heterotrait-monotrait (HTMT) ratio criterion]. Multiple logistic regression analyses tested associations between patient/surgical characteristics and various factors of the Arabic questionnaire.

**Results:**

Exploratory factor analysis generated a three-factor solution (18 items): ‘concerns’ about shape/weight/eating (9 items), ‘restraint’ behaviors (4 items), and ‘purging’ behaviors (5 items), explaining 22% of the total variance. Confirmatory factor analysis confirmed this factor structure displayed good model-data fit, with comparative fit index (0.96) and Tucker–Lewis index (0.95) both > 0.95 threshold; *χ*^2^/df ratio = 1.52 (recommended value ≤ 2); root mean square error of approximation = 0.031 (90%CI:0.022–0.040, *p* = 1.000) and standardized root mean square residual = 0.047 (recommended values ≤ 0.05). Cronbach’s alpha (internal consistency) was 0.80 for ‘concerns’ (95%CI:0.78–0.82), 0.62 for ‘restraint’ (95%CI:0.55–0.68), and 0.61 for ‘purging’ behavior (95%CI:0.51–0.69). EDABS-Q-Arabic18’s discriminant validity was excellent, confirming the distinctiveness of each factor, with 0.208 HTMT ratio between ‘concerns’ and ‘restraint’ factors, 0.198 between ‘concerns’ and ‘purging’, and 0.257 between ‘restraint’ and ‘purging’ factors (recommended thresholds < 0.85–0.90). The prevalence of ‘concerns’ was 98.4%, with patients experiencing mild (31.1%), moderate (48.9%), severe (18.4%), or no (1.6%) concerns. The prevalence of ‘restraint’ behaviors was high (79.7%) but mostly mild (53.1%) or moderate (22.5%). ‘Purging’ behaviors had a lower prevalence (44.2%), with 40.1% mild, 3.8% moderate, and 0.3% severe purging. Logistic regression showed that for severe ‘concerns’, increasing age and pre-operative BMI displayed lower odds OR = 0.98, 95%CI 0.96–1.00, *p* = 0.013; OR = 0.96, 95%CI 0.93–0.98, *p* = 0.001 respectively), while current BMI and time since surgery exhibited significant positive associations (OR = 1.19, 95%CI 1.14–1.24, *p* < 0.001; OR = 1.10, 95%CI 1.02–1.18, *p* = 0.011, respectively). For severe ‘restraint’ behaviors, only pre-operative BMI displayed significant association (OR = 1.05–95%CI 1.00–1.09, *p* = 0.023). Moderate to severe ‘purging’ behaviors had no significant associations with any patient/surgical characteristics. The type of MBS procedure was not associated with any of the three factors.

**Conclusions:**

The 18-item Arabic version of the Eating Disorder Examination-Self-report Questionnaire Bariatric Surgery Version (EDABS-Q-Arabic18) is a culturally appropriate, valid, and reliable tool for assessing post-MBS ED. Further validation across Arabic-speaking populations is encouraged to strengthen its broader applicability.

**Supplementary Information:**

The online version contains supplementary material available at 10.1007/s11695-025-07910-9.

## Introduction

Metabolic and bariatric surgery (MBS) introduces new anatomo-functional gastrointestinal conditions and resulting modifications in the entero-hormonal pattern. These lead to changes in food transit, digestion and absorption, and appetite/satiety balance, with consequent alterations in eating behavior [1]. Such alterations include eating disorders (ED), defined as medical illnesses that produce disturbances to one’s eating behaviors, diagnosed by a range of psychological, behavioral, and physiological characteristics [2]. Even more widespread are disordered eating behaviors (DEB), defined as problems related to eating, including purgative practices, compulsions, food restriction, and other inappropriate methods to lose or control weight that occurs less frequently or less severely than required by the diagnostic criteria of ED [3, 4].

After MBS, patients are at risk of development or recurrence of DEB and ED. These conditions are negative predictors of surgical weight loss (WL) and its maintenance, and are associated with significant morbidity, mortality, and poor psychosocial functioning [5–8]. ED are more common among MBS candidates than the general population [8], and although ED symptoms are present in many post-MBS patients [9], these cases are frequently not reported due to their sub-syndrome manifestation.

ED after MBS have generally not been appraised in the Middle East and North Africa (MENA) region [10]. A study in Bahrain recruited a modest sample of 48 patients and examined ED behaviors related to sleeve gastrectomy [10]; and a second in Saudi Arabia assessed the relationship between obesity, eating disorders, and physical health, but only 7% of the participants had undergone MBS [11]. Both studies noted that more research is needed. Certainly, more inquiries of post-MBS ED are required across MENA for several reasons.

The first is the prevalence of obesity in MENA, where adults with overweight or obesity and metabolic syndrome amount from 25 to 82% and 24 to 40%, respectively [12, 13], suggesting that non-surgical and surgical WL interventions are required across MENA. Second is that (nonsurgical) dietary interventions appear not to be popular in MENA and demonstrate limited effectiveness (mean WL = 4.8 kg) [14]. Lifestyle and pharmacological weight management are frequently unsatisfactory, outcomes are not maintained in the longer term, and older WL pharmacotherapy exhibits side effects [15–17]. Third is the increase in MBS globally and in MENA [18–21]. In Saudi Arabia, about a third of 31–40-year-olds have had MBS [22], and government insurance includes MBS coverage in some MENA nations [18]. Although MBS results in remarkable short- and long-term WL, and resolution of associated medical conditions [23–25], however, weight recurrence is not uncommon across MENA [26–29]. Such recurrence could precipitate ED.

Instruments that assess eating pathology among MBS populations are limited [30]. Investigations of DEB and ED among adults seeking MBS traditionally relied on structured interviews [31], considered the ‘gold standard’ [32], e.g., the Eating Disorder Examination-Bariatric Surgery Version (EDE-BSV) [33]. However, interviews require resources, experienced interviewer/s, and significant time. Self-reported questionnaires are scarce. One that has been recently published (EDABS-Q) assessed ED after MBS without the need for an interview [34]. However, there is no culturally adapted self-administered Arabic language tool to assess ED among MBS patients across MENA, despite the demand for such a tool.

The literature reveals knowledge gaps in relation to MENA. For instance, the two ED studies in MENA used non-Arabic language questionnaires [35, 36] but did not clarify whether these were translated to Arabic and culturally adapted, nor provided their psychometric performances [10, 11]. A few other published instruments appraised related issues e.g., general nutrition knowledge of Arabic-speaking MBS patients, but no psychometric data were presented [37–40]. Conversely, others presented psychometric data for their tools used among pre-/post-MBS patients, but these were not mainly for the appraisal of ED e.g., Quality-of-Life and Eating Behavior After Bariatric Surgery questionnaires [41, 42].

To the authors’ knowledge, to date, there is no Arabic language questionnaire that appraises eating pathology after MBS without an interview. This is despite that culturally congruent care is more effective [43]. The current study bridged this knowledge gap. The aim was to translate the self-administered English-published version of the validated Eating Disorder Examination-Self-report Questionnaire Bariatric Surgery Version (EDABS-Q) [34] into Arabic (EDABS-Qarabic), culturally adapt it, and assess its psychometric properties. The specific objectives were to a) translate the EDABS-Q into Arabic (EDABS-Q-Arabic) and culturally adapt it to MENA populations; b) randomly select 1100 patients who had MBS since ≥ 1 year to complete the Arabic questionnaire (EDABS-Q-Arabic); c) assess the questionnaire’s face validity (expert panel); d) appraise the questionnaire’s construct validity (exploratory factor analysis, confirmatory factor analysis, structural model); e) evaluate the questionnaire’s internal consistency (internal reliability analysis, and f) gauge the questionnaire’s discriminant validity (heterotrait-monotrait ratio criterion). Moreover, the study aimed to provide insights into practical tools for clinicians to identify patients likely to exhibit ED. Hence, another two objectives were to g) appraise the prevalence and severity of the factors of the Arabic eating disorders questionnaire across the sample and, h) explore the features associated with each factor of the questionnaire, using a range of patient demographic and pre-/post-operative anthropometry features, as well as surgical characteristics.

Given that there are no self-administered Arabic-language tools to appraise post-MBS ED, the findings of this study are timely, providing a practical and time-efficient alternative for clinical and research settings. A comprehensive understanding of post-MBS eating pathology in MENA, their dimensions, and their relationships with operative and patient features are critical for bariatric teams, patients and families, hospital administrators, and other stakeholder groups across Arabic-speaking countries and cultures.

## Material and Methods

### Study Design, Ethics and Inclusion Criteria

The Ethics Committee of Alexandria University, Egypt approved this cross-sectional study. The study was carried out in accordance with the Helsinki Declaration. Informed Consent Forms were signed by all participants who agreed to participate. Inclusion criteria were (i) adult patients aged > 18 years old, (ii) both sexes, (iii) previous MBS procedure at least before 2023, (iv) any kind of MBS, (v) could read Arabic, and (vi) agreed and consented to participate.

### Sample Size

To explore the construct validity of the Arabic EDABS questionnaire, we selected a sample size of ≥ 1000 patients of both sexes who had undergone MBS for ≥ 1 year, as previous recommendations of a sample size required for scale development of ≥ 1000 is graded as excellent [44]. Hence, we aimed to sample 1200 patients, providing a 20% extra for rejections.

### Questionnaire Translation and Adaptation: Stages

#### Permission

At the beginning of the study, permission was received from the developer of the survey [34]. The adaptation process was conducted in accordance with published guidelines [45].

#### Forward Translation

Two forward translations were undertaken by two authorized bilingual translators, one acquainted with the construct of the research, while the other was unaware. The published English version (EDABS-Q) was translated into Arabic (EDABS‑Q‑Arabic). The translation process was straightforward, with only minor challenges encountered. Accuracy was verified, and adjustments were made as necessary to ensure linguistic and cultural equivalence and appropriateness.

#### Synthesis

Translations that were generated were verified and converted into a single translation.

#### Backward Translation

The generated EDABS‑Q‑Arabic was translated back to English by two native English-speaking translators who were unaware of the original (EDABS-Q) version and without medical expertise. Two English back translations were achieved.

#### Expert Panel

A bilingual expert board, proficient in English and Arabic, meticulously appraised the original EDABS-Q and the translations, with attention to semantic, idiomatic, experiential, and conceptual equivalences. The board also assessed the face and content validity of the questionnaire in addressing the constructs under examination, with consideration of the clarity, comprehensibility, and linguistic and cultural nuances specific to the Arabic-speaking populations. The EDABS‑Q‑Arabic was finalized.

#### Prefinal Version

The final EDABS‑Q‑Arabic generated was administered and tested on 30 post-MBS patients who were excluded from the final sample. Aspects pertaining to the meaning of the items and the patients’ responses were assessed.

### Participants and Data Collection: Questionnaire

**Fig. 1:**
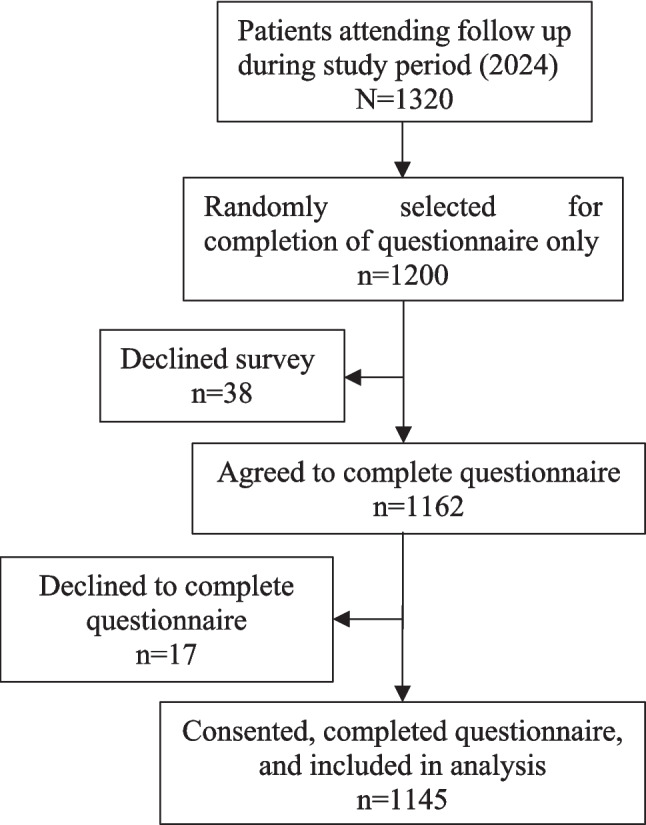
Flow chart of patients

Figure 1 depicts the recruitment of participants for the study. Patients were approached while waiting in the clinic for their follow-up appointment with the bariatric team. A member of the research team explained the aims and objectives of the research to all patients and invited them to participate.

After their bariatric consultation, those who wished to participate signed an informed consent and were provided with a paper Arabic questionnaire to complete. The same member of the research team collected the completed questionnaires, and respondents with incomplete responses were encouraged to complete the responses to the questionnaire to the best of their ability. Completion of the questionnaire required about 30 min. No incentives or compensation were provided for the completion of the questionnaire.

The initial questionnaire comprised 41 items. These were categorized into three factors, namely, ‘concerns’ about shape, weight, and eating (17 items), ‘restraint’ behaviors for weight control and/or to avoid physical discomfort (10 items), and ‘purging’ behaviors for weight control and/or to avoid physical discomfort (14 items). Exploratory factor analysis resulted in condensing the 41 items to 18 items that were tested by confirmatory factor analysis. This generated the final EDABS-Q-Arabic18.

### Statistical Analysis

Categorical variables were presented as frequencies and percentages, while continuous variables were presented as means and standard deviations.

Construct validity was assessed using exploratory factor analysis (EFA) and confirmatory factor analysis (CFA). As recommended, the data set was randomly split into two as conducting both exploratory and confirmatory factor analysis on the same set of data leads to overfitting [46]. Initially, the data set (*n* = 1145) was randomly split into two equal parts. Next, EFA (data set 1, *n* = 573) and CFA (data set 2, *n* = 572) were performed. No missing responses were found for the Arabic EDABS questionnaire for both the EFA and CFA datasets.

The multivariate normal distribution of the EFA dataset was examined using Mardia’s skewness and kurtosis measurements, which generated values of 95.22 (*p* < 0.001) and 41,323.24 (*p* < 0.001), respectively. Factors were extracted using principal axis factoring (PAF) as the multivariate normality assumption was not met. For multivariate outliers, Mahalanobis distance was employed. We removed the 43 multivariate outliers in the EFA sample, excluding them from further analysis. For the EFA, we employed the Pearson product-moment (PPM) correlation matrix, as when there are five or more categories of variables, variables can be accepted as continuous [47]. Kaiser–Meyer–Olkin (KMO) test assessed sample adequacy, required to be ≥ 0.50 [48]; and Bartlett’s test of sphericity [49] evaluated data factorability to ensure the correlation matrix was not an identity matrix. Bartlett’s sphericity test was statistically significant (χ^2^(820) = 5286.4, *p* < 0.001), and the KMO value was 0.77, confirming that the data was factorable and the sample size was adequate.

Kaiser criterion (factors with eigenvalues > 1) was examined for factor retention [48]. The Scree test (number of factor points above the break and excluding the breakpoint), optimal parallel analysis (PA), minimum average partials (MAP), and percentage of variance criterion (the solution that accounts for at least 60%) were appraised as the Kaiser criterion might overestimate the number of factors. EFA was conducted for seven dimensions as the adapted scale (EDABS) is seven-dimensional.

Due to the ordinal nature of the 41 items, we used an iterated principal axis factoring for factor extraction, which is preferred for such scales [50]. The construct was developed assuming its factors would be correlated (Supplementary Appendix I); therefore, we used “direct oblimin” rotation. A first-order CFA was used to confirm the decided factor structure resulting from the EFA results. For model evaluation and selection of the final model structure, we examined several model fit statistics: model chi-square, comparative fit index (CFI), Tucker–Lewis index (TLI), root mean square error of approximation (RMSEA), and standardized root mean square residual (SRMR). The acceptable fit was indicated when *χ*^2^/df < 5 [51], CFI > 0.90 [52, 53], TLI > 0.90 [53], SRMR < 0.08 [54], and for RMSEA, a good fit is proved when ≤ 0.05 or 90% CI including 0.05, and *p* > 0.05 meant that the RMSEA test is not significant and not rejecting the null hypothesis that the RMSEA is ≤ 0.05 [55].

For the CFA dataset, Mardia’s skewness and kurtosis values were 39,187.98 (*p* < 0.001) and 80.7 (*p* < 0.001), respectively. For parameter estimation, the maximum likelihood robust (MLR) estimation approach was applied as the multivariate normality requirement was not upheld. Multivariate normal distribution violations can be effectively mitigated using MLR [56, 57]. We removed 42 multivariate outliers in the CFA sample from the CFA analysis based on Mahalanobis distance for multivariate outliers. Cronbach’s alpha confirmed the internal consistency of each factor identified in the measurement model, and, in line with others, internal validity was considered when Cronbach’s alpha was > 0.6 [58].

Discriminant validity was assessed using the heterotrait-monotrait (HTMT) ratio of correlations to examine the distinctiveness of the factors in the EDABS-Q-Arabic. The HTMT ratio was calculated based on the multitrait-multimethod matrix, with values < 0.85 considered indicative of adequate discriminant validity. The analysis was performed using semTools package in R [59, 60].

The final model served as the basis for calculating category scores. Items comprising each factor were averaged, and the resulting averages were utilized to categorize symptom categories based on: 0 = none, > 0 to < 3 = mild, 3 to < 5 = moderate, and ≥ 5 = severe. Subsequently, the prevalence of each level was determined, and multiple logistic regression analyses assessed the impact of patient characteristics on the probabilities of experiencing severe symptoms.

Software R was utilized for the analysis [61]. We employed psych (version 2.2.5) [62] for KMO and Bartlett’s test, and code written for testing assumptions [63]. The psych [62] and lavaan (version 0.6–12) [64] packages were used for EFA and CFA, respectively. Cronbach’s alpha was obtained using the psych package [62]. The alpha criterion for statistical significance was set at *p* = 0.05.

## Results

### General Characteristics of the Sample

A total of 1145 patients completed the survey. The sample’s mean age was 39.3 ± 9.5 years, with the majority females (82.4%) (Table 1). Average time since MBS was 3.4 years. The most common MBS was non-ringed augmented laparoscopic sleeve gastrectomy (85.8%), followed by non-ringed one anastomosis gastric bypass and ringed augmented sleeve gastrectomy. Most procedures were primary (98.4%) with fewer revisional surgeries (1.6%).

**Table 1:** Characteristics of the sample (*N*=1145)

Characteristic	Value
Demographics	
Age (years)	39.6 ± 9.5 (18–71)
Gender ^*a*^	
Female	943 (82.4)
Male	202 (17.6)
Surgery	
Time since MBS (years)	3.4 ± 2.1 (1–12)
Type ^*a*^	
Non-ring augmented	
Sleeve gastrectomy	982 (85.8)
One anastomosis gastric bypass	124 (10.8)
Roux-en-Y gastric bypass	13 (1.1)
Ring-augmented	
Sleeve gastrectomy	23 (2.0)
One anastomosis gastric bypass	3 (0.3)
Order ^*a*^	
Primary	1127 (98.4)
Revision	18 (1.6)
Anthropometry	
Preoperative values	
Height (cm)	166.0 ± 8.1 (145.0–197.0)
Weight (kg)	131.8 ± 26.2 (64.0–260.0)
BMI (kg/m^2^)	47.7 ± 8.1 (23.8–92.6)
Best anthropometric values	
Nadir weight (kg)	76.8 ± 14.8 (44.0–145.0)
Maximum weight lost (kg)	55.0 ± 19.6 (7.0–175.0)
Nadir BMI (kg/m^2^)	27.9 ± 4.9 (15.6–59.6)
%EWL	76.5 ± 14.9 (12.4–133.3)
%TWL	41.0 ± 8.6 (8.2–72.7)
Current values (at time of study)	
Weight (kg)	83.7 ± 16.8 (45.0–180.0)
BMI (kg/m^2^)	30.4 ± 5.6 (17.1–63.8)
%EWL	66.5 ± 17.1 (0.0–125.9)
%TWL	35.8 ± 9.8 (0.0–66.9)
Weight recurrence	
Amount (kg)	6.9 ± 8.6 (0.0–87.0)
% of sample with WR ^*a*^	287 (25.1)
% WR from max weight lost ^*a*^ (kg)	13.0 ± 15.0 (0.0–100.0)

Cell values represent mean ± SD (minimum-maximum) unless otherwise indicated; ^*a*^ Cell values represent frequency (%); *MBS* metabolic and bariatric surgery; *max* maximum; *BMI* body mass index; *%EWL* percentage excess weight loss; *%TWL* percentage total weight loss; *WR* weight recurrence (≥ 20% weight regain from maximum weight lost)

Preoperatively, the mean weight was 131.8 kg, and the mean body mass index (BMI) was 47.7 kg/m^2^. After surgery, there was a significant reduction in weight, reaching a maximum WL of 55 kg and mean nadir weight of 76.8 kg, corresponding to a mean nadir BMI of 27.9 kg/m^2^, with percent excess weight loss (%EWL) and percent total weight loss (%TWL) averaging 76.5 and 41.0%, respectively. At the time of the study, the mean weight was 83.7 kg, and BMI was 30.4 kg/m^2^. A quarter (25.1%) of respondents experienced weight recurrence, and the mean weight recurrence was 6.9 kg, equating to a mean 13% weight regain from maximum WL.

### Questionnaire: Post-MBS Eating Disorders

The responses to the items of EDABS-Q-Arabic are depicted in Table 2. In terms of the ‘concerns’ factor, there were significant concerns related to shape, weight, and eating behaviors. Items within the shape sub-category demonstrated dissatisfaction with body shape and fears of weight gain, indicated by mean scores between 2.7 and 5.0 on the 7-point scale. Similarly, in the weight sub-category, respondents felt dissatisfaction with weight and strong desires for WL, reflected by scores between 1.6 and 5.3. Pertaining to the eating sub-category, individuals reported difficulties concentrating due to food intake and out-of-control eating.

**Table 2:** Items (*n* = 41) of the Eating Disorders After MBS Arabic version questionnaire (EDABS-Qab) used in the exploratory factor analysis

**Factor** ^*a*^	**Item**	**Prompt**	**Mean ± SD**
**‘Concerns’ about**			
Shape	13	Days desiring flat stomach	4.4 ± 2.4
	15	Difficulty concentrating due to shape or weight concern	3.2 ± 2.7
	17	Fear of weight gain	5.0 ± 2.0
	18	Feeling fat	4.4 ± 2.3
	79	Shape influenced self-judgment	3.6 ± 2.3
	82	Dissatisfaction with shape	2.7 ± 2.3
	83	Uncomfortable seeing body	2.9 ± 2.3
	84	Uncomfortable having others see body	2.6 ± 2.4
Weight	19	Strong weight loss desire	5.3 ± 1.8
	78	Weight influenced self-judgment	3.6 ± 2.3
	80	Upset if asked to weigh self	1.6 ± 2.1
	81	Dissatisfaction with weight	3.1 ± 2.4
Eating	14	Difficulty concentrating due to food intake	1.9 ± 2.5
	16	Days fearing out of control eating	4.3 ± 2.5
	75	Days eating in secret	0.9 ± 1.7
	76	Proportion of times felt guilty	2.9 ± 2.4
	77	Concern over other people seeing you eat	1.3 ± 1.9
**‘Restraint’ behavior**			
For weight control	1	Days limiting food to avoid weight gain	3.3 ± 2.5
	3	Days without eating to avoid weight gain	1.3 ± 2.0
	5	Days excluded liked foods to avoid weight gain	3.0 ± 2.5
	8	Follow diet rules to avoid weight gain	2.4 ± 2.5
	11	Days desiring empty stomach to avoid weight gain	2.1 ± 2.5
To avoid physical discomfort	2	Days limiting food to avoid physical discomfort	3.2 ± 2.6
	4	Days without eating to avoid physical discomfort	1.0 ± 1.9
	6	Days excluded liked foods to avoid physical discomfort	2.7 ± 2.6
	9	Follow diet rules to avoid physical discomfort	2.0 ± 2.5
	12	Days desiring empty stomach to avoid physical discomfort	1.9 ± 2.4
**‘Purging’ behavior**			
For weight control	46	Vomit to lose weight or avoid weight gain	0.5 ± 1.2
	48	Days purposely chewed food and spit it out without swallowing to lose weight/avoid gaining weight	0.3 ± 0.9
	50	Upset after chewing food or spitting it out without swallowing	1.0 ± 1.9
	63	Days ruminated food to lose weight or avoid gaining weight	0.2 ± 0.8
	65	Upset when ruminated food	1.0 ± 2.0
	66	Take laxatives to lose weight or avoid gaining weight	0.3 ± 1.0
	68	Take diuretics to lose weight or avoid gaining weight	0.1 ± 0.7
	70	Driven or compulsive exercise to lose weight	0.9 ± 1.6
	85	Vomited in last 4 weeks, how upset about it	1.3 ± 2.1
To avoid physical discomfort	47	Vomit to avoid physical discomfort	0.8 ± 1.4
	49	Days purposely chewed food and spit it out without swallowing to avoid physical discomfort	0.4 ± 1.0
	64	Days ruminated food to avoid physical discomfort	0.3 ± 1.0
	67	Take laxatives to avoid physical discomfort	0.4 ± 1.1
	69	Take diuretics to avoid physical discomfort	0.1 ± 0.5

^*a*^ Categories from the adapted EDABS. Mean scores and standard deviations provided for each item based on 7-point Likert scale that represents frequencies of behaviors (reported in days, 0–28), converted into 7-point ordinal scale, where: “0” = 0 days; “1” = 1–5 days s; “2” = 6–12 days s; “3” = 13–15 days s; “4” = 1 6–22 days; “5” = 23–27 days; “6” = everyday

As for the ‘restraining’ factor, behaviors related to restraint for weight control and to avoid physical discomfort were evident, with mean scores indicating moderate levels of engagement in restrictive eating behaviors.

Regarding the ‘purging’ factor, behaviors pertaining to purging for weight control and to avoid physical discomfort were reported infrequently, albeit still present.

### Exploratory Factor Analysis

**Fig. 2:**
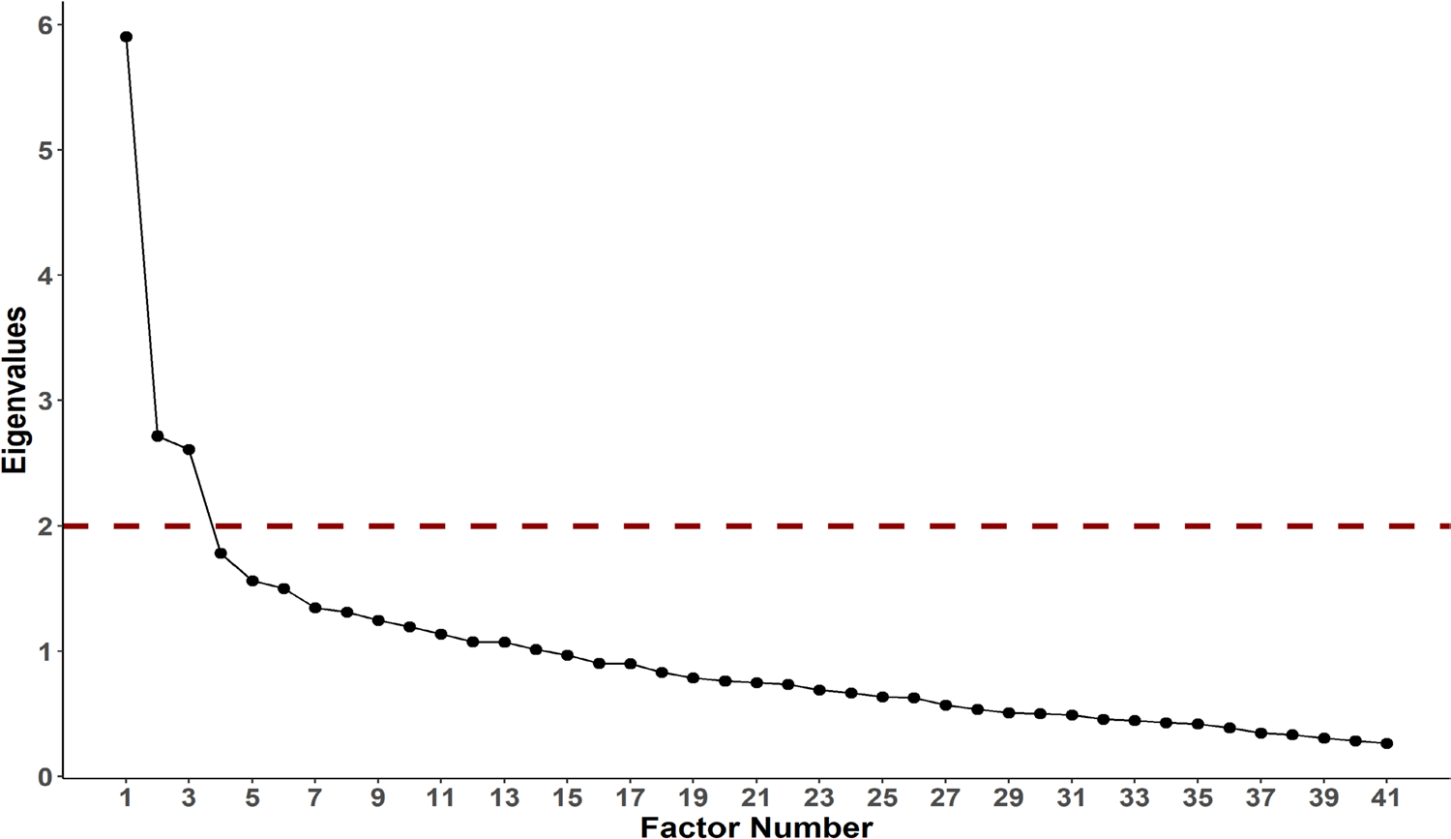
Scree plot

EFA showed that the first 14 eigenvalues were > 1, and the remaining 27 were < 1. Therefore, based on the Kaiser criterion, the number of dimensions was 14 (Fig. 2). The percentage of variance criterion also recommended 14 factors that accounted for ≥ 60% of the total variance. Optimal parallel analysis recommended 9 factors while both MAP and the Scree test recommended three factors (Fig. 1). Examining the factor loadings of the 14- and 9-factor solutions suggested that the Kaiser criterion, the percentage of variance criterion, and PA overestimated the number of factors as several factors had only one or two items with loadings ≥ 0.4, and items belonging to the same category in the original EDABS were loaded on three or four factors (Supplementary Appendices II, III). Likewise, the 7-factor solution also overestimated the number of factors (Supplementary Appendix IV). Hence, the three-factor solution was selected.

Table 3 depicts the selected three-factor solution. In terms of the ‘concern’ domain, items belonging to weight, shape, and eating concerns dimensions in the original EDABS were highly loaded on the same factor (PA1), and only those with loadings ≥ 0.4 that were kept in the model (Q17, Q18, Q82, Q83, Q84, Q80, Q81, Q76, and Q77). Regarding the ‘restraint’ factor, all items belonging to both restrain for weight control and to avoid physical discomfort were highly loaded on the same factor (PA2) except Q8, which was deleted (loading < 0.4), leaving Q1, Q3, Q5, Q11, Q2, Q4, Q6, Q9, and Q12 in the model. As for the ‘purging’ factor, five items belonging to the two original purging dimensions (Q48, Q50, Q63, Q65, and Q49) were merged into one dimension as they loaded on one factor (PA3), while the remaining purging items were not highly loaded on any factor.

**Table 3:** Exploratory factor analysis of the three-factor solution

Factor ^a^	Item	PA1	PA2	PA3	*h*^*2*^
**‘Concerns’ about**					
Shape	13				0.20
	15				0.21
	17	0.41			0.36
	18	0.51			0.33
	79				0.11
	82	0.67			0.45
	83	0.69			0.46
	84	0.59			0.34
Weight	19				0.28
	78				0.12
	80	0.41			0.19
	81	0.69			0.48
Eating	14				0.11
	16				0.31
	75				0.12
	76	0.48			0.25
	77	0.47			0.26
‘**Restraint’ behavior**					
For weight control	1		0.42		0.26
	3		0.47		0.23
	5		0.51		0.29
	8				0.15
	11		0.47		0.28
To avoid physical discomfort	2		0.47		0.22
	4		0.49		0.27
	6		0.57		0.30
	9		0.46		0.20
	12		0.52		0.30
**‘Purging’ behavior**					
For weight control	46				0.12
	48			0.40	0.19
	50			0.42	0.22
	63			0.50	0.29
	65			0.42	0.19
	66				0.06
	68				0.13
	70				0.02
	85				0.13
To avoid physical discomfort	47				0.08
	49			0.40	0.17
	64				0.17
	67				0.09
	69				0.10
Proportion of variance explained		10%	8%	5%	
Total variance explained			22%		
Inter-factor correlations		PA1	PA2	PA3	
	PA1	1.00			
	PA2	0.28	1.00		
	PA3	0.04	0.05	1.00	

^*a*^ Original categories from the adapted EDABS. Only factor loadings ≥ 0.4 are presented. *PA*, pattern matrix’s factor loadings; *h*^*2*^, communalities

### Confirmatory Factor Analysis

A CFA was conducted to validate the factor structure identified in the EFA. The initial model demonstrated poor fit for some indices and acceptable fit for others. Specifically, the CFI (0.86) and TLI (0.87) indicated poor fit, as they were below the recommended threshold of 0.90**.** The *χ*^2^/df ratio (2.35) suggested an acceptable fit (with values between 1 and 3 being considered acceptable). The RMSEA (0.051; 90% CI: 0.04–0.06, *p* = 0.443) did not indicate either a good or poor fit (with values ≤ 0.05 considered good, while values ≥ 0.1 indicating poor fit), and the SRMR (0.055) also reflected acceptable fit (with values between 0.05 and ≤ 0.08 being acceptable).

**Fig. 3:**
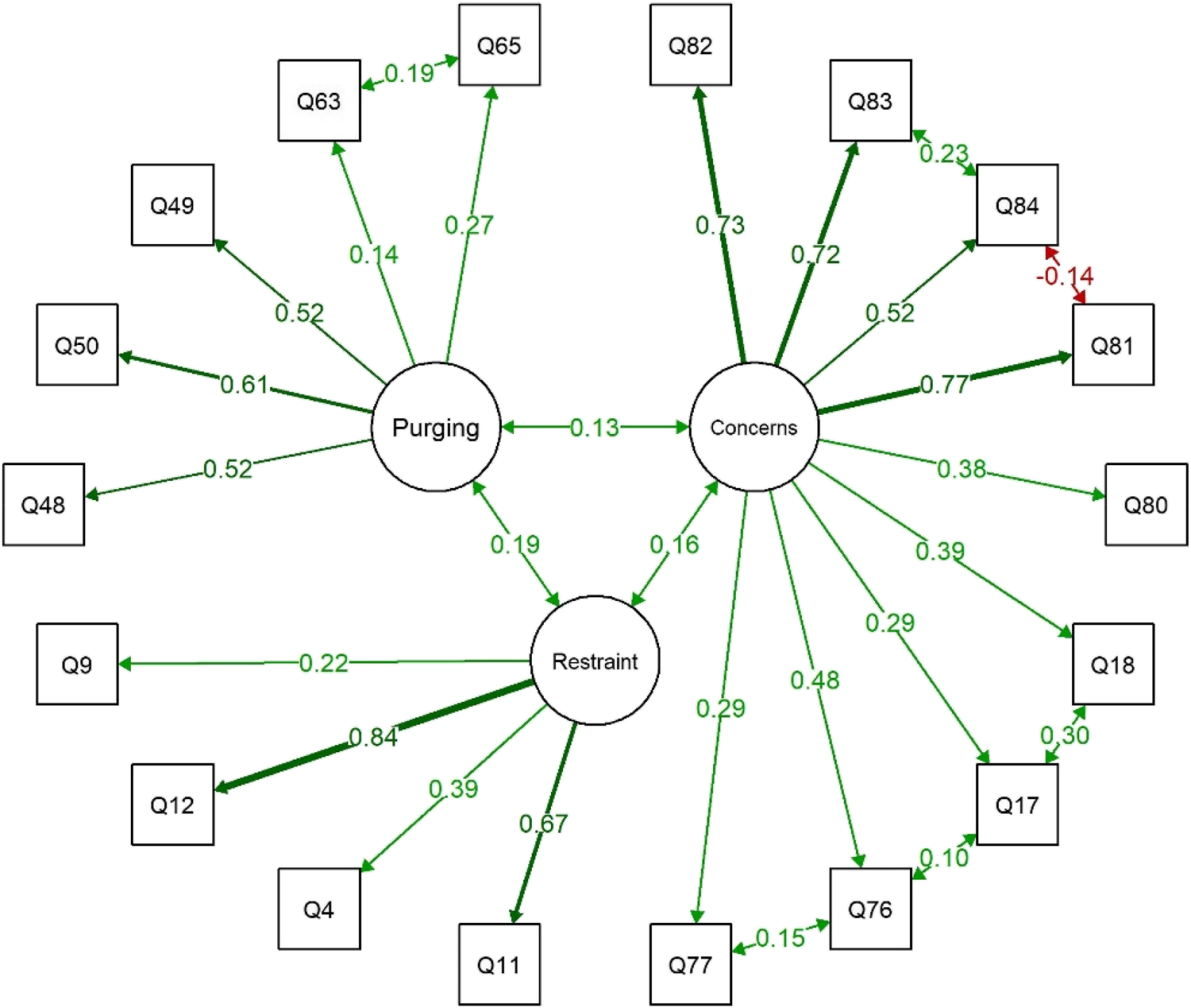
Structural model from the confirmatory factor analysis of the Eating Disorders After Bariatric Surgery Arabic Questionnaire (EDABS-Q-Arabic18). Note: The structural model derived from confirmatory factor analysis (CFA) for EDABS-Q-Arabic18, presenting standardized path coefficients for the 3-factor structure: ‘concerns’ about shape/weight/eating (*α* = 0.80), ‘restraint’ behavior (*α* = 0.62), and ‘purging’ behavior (*α* = 0.61). Model fit indices indicated acceptable fit (*χ*^2^/df = 1.52, CFI = 0.96, TLI = 0.95, RMSEA = 0.031, SRMR = 0.047). Significant standardized coefficients (*p* < 0.05) demonstrate relationships between latent variables and their respective items, as well as covariances among specific items.

Examination of the modification indices revealed high indices, ranging between 12.7 and 47.5, for six pairs of items (Q83/Q84, Q63/Q65, Q84/Q81, Q76/Q77, Q76/Q17, and Q18/Q17). To improve model fit, covariances between error terms were added for these six pairs. The revised model exhibited a good fit across all indices. Specifically, the CFI (0.96) and TLI (0.95) indicated a good fit as they were above the recommended threshold of 0.95. The *χ*^2^/df ratio (1.52) also suggested a good fit (values ≤ 2 considered good). RMSEA (0.031; 90% CI: 0.022–0.040, *p* = 1.000) demonstrated good fit (values ≤ 0.05 indicating good fit), and the SRMR (0.047) also demonstrated good fit (≤ 0.05). The final structural model for the EDABS-Qab is presented in Fig. 3 and Table 4.

**Table 4:** Confirmatory factor analysis for final items (*n*=18) of Eating Disorders after MBS Arabic questionnaire (EDABS-Q-Arabic18)

Factor and Items	Coefficient	SE	Std Coefficient	*p*
**‘Concerns’ about shape/weight/eating**	**Cronbach's α**=0.80 (95% CI: 0.78–0.82)			
Q17: Fear of weight gain	0.59	0.09	0.29	*< 0.001*
Q18: Feeling fat	0.93	0.10	0.39	*< 0.001*
Q82: Dissatisfaction with shape	1.70	0.08	0.73	*< 0.001*
Q83: Uncomfortable seeing body	1.66	0.08	0.72	*< 0.001*
Q84: Uncomfortable having others see body	1.25	0.10	0.52	*< 0.001*
Q80: Upset if asked to weigh self once	0.82	0.10	0.38	*< 0.001*
Q81: Dissatisfaction with weight	1.81	0.08	0.77	*< 0.001*
Q76: Proportion of times felt guilty	1.15	0.10	0.48	*< 0.001*
Q77: Concern over other people seeing you eat	0.50	0.08	0.29	*< 0.001*
**‘Restraint ‘behavior**	**Cronbach's α**=0.62 (95% CI: 0.55–0.68)			
Q11: Days desiring empty stomach to avoid weight gain	1.68	0.14	0.67	*< 0.001*
Q4: Days w/o eating to avoid physical discomfort	0.74	0.11	0.39	*< 0.001*
Q9: Follow diet rules to avoid physical discomfort	0.55	0.13	0.22	*< 0.001*
Q12: Days desiring empty stomach to avoid physical discomfort	2.03	0.15	0.84	*< 0.001*
**‘Purging’ behavior**	**Cronbach's α**=0.61 (95% CI: 0.51–0.69)			
Q48: Chewed food/spit without swallowing to lose weight/avoid	0.32	0.06	0.52	*< 0.001*
Q50: Upset after chewing food or spitting it out without swallowing	1.16	0.15	0.61	*< 0.001*
Q63: Days ruminated food to lose weight or avoid gaining weight	0.06	0.03	0.14	*0.045*
Q65: Upset when ruminated food	0.51	0.13	0.27	*< 0.001*
Q49: Chewed food/spit without swallowing to avoid physical discomfort	0.34	0.06	0.52	*< 0.001*
**Covariances**				
Q83~~Q84	0.73	0.25	0.23	*0.003*
Q63~~Q65	0.16	0.04	0.19	*< 0.001*
Q84~~Q81	-0.43	0.20	–0.14	*0.032*
Q76~~Q77	0.52	0.17	0.15	*0.002*
Q17~~Q76	0.40	0.17	0.10	*0.020*
Q18~~Q17	1.23	0.23	0.30	*< 0.001*
Concerns~~Restraint	0.16	0.05	0.16	*0.002*
Concerns~~Purging	0.13	0.06	0.13	*0.040*
Restraint~~Purging	0.19	0.07	0.19	*0.008*
**Fit indices**				
χ2/df	1.52			
Comparative fit index	0.96			
Tucker Lewis index	0.95			
Root mean square error of approximation	0.031			
Standardized root mean square residual	0.047			

*Q* question number; *SE* standard error; *Std* standard; α alpha; *CI* confidence interval; italicized cells indicate statistical significance

### Internal Consistency

Table 4 also shows that the Cronbach’s alpha internal consistency statistic computed for each factor were all > 0.6. For the ‘concerns’ about weight, shape, and eating, it was 0.80 (95% CI: 0.78–0.82), and for the ‘restraint’ behavior factor and ‘purging’ behavior factor, it displayed 0.62 (95% CI: 0.55–0.68) and 0.63 (95% CI: 0.51–0.69), respectively.

### Discriminant Validity

Table 5 presents the results of the HTMT ratio analysis used to assess discriminant validity. The results demonstrated that the HTMT ratios between the factors were all well below the recommended thresholds of 0.85 and 0.90, indicating that each of the factors is distinct. Specifically, the HTMT ratio was 0.208 between ‘concerns’ and ‘restraint’ behaviors factors, 0.198 between ‘concerns’ and ‘purging’ behaviors factors, and 0.257 between ‘restraint’ and ‘purging’ behaviors factors. These findings strongly supported the discriminant validity of the EDABS-Q-Arabic18 constructs.

**Table 5:** Discriminant validity: heterotrait-monotrait ratios of Eating Disorders after MBS Arabic questionnaire (EDABS-Q-Arabic18) factors

Factor	‘Concerns’ about shape/weight/eating	‘Restraint’ behavior	‘Purging’ behavior
‘Concerns’ about shape/weight/eating (nine items)	1.000		
‘Restraint’ behavior (four items)	0.208	1.000	
‘Purging’ behavior (five items)	0.198	0.257	1.000

### Prevalence of Eating Disorders After MBS

**Fig. 4:**
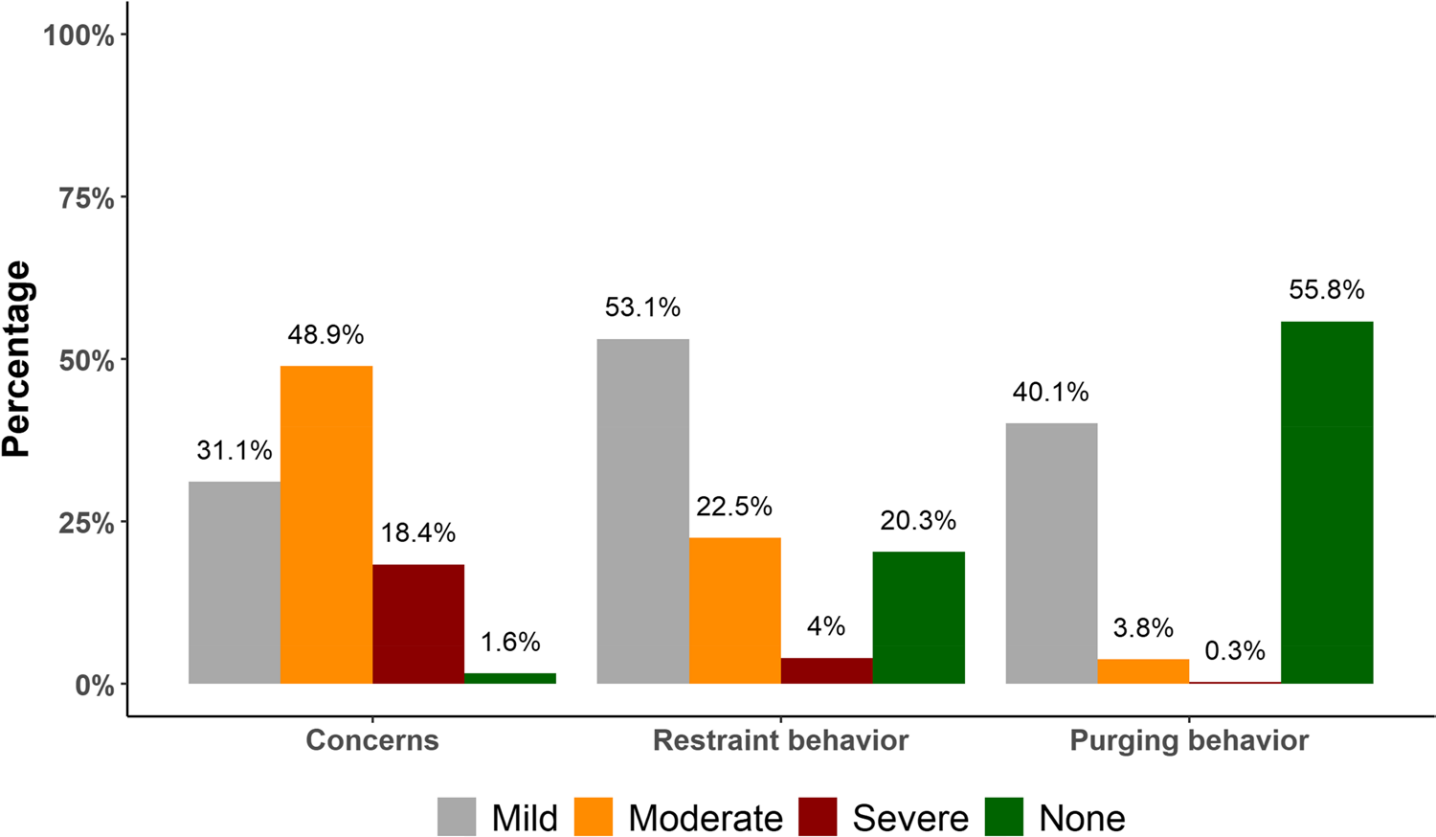
Prevalence of eating disorders after MBS among the sample

Table 6 and Fig. 4 depict the prevalence and severity of eating disorders after MBS, in terms of ‘concerns’ about shape/weight/eating, ‘restrain’ behavior, and ‘purging’ behavior. The mean ‘concerns’ score was 2.9 ± 1.5, indicating moderate concern on average among the sample. The ‘concerns’ prevalence was notably high, with 98.4% of individuals experiencing some degree of shape/weight/eating concerns. The distribution of severity levels across the series was as follows: mild (31.1%), moderate (48.9%), severe (18.4%) concerns, and no concerns (1.6%).

**Table 6:** Prevalence of eating disorders after MBS using the Arabic questionnaire (EDABS-Q-Arabic18) (*n* = 1145)

Variable	Value
Shape/weight/eating concerns	
Score, mean ± SD	2.9 ± 1.3
Prevalence	1127 (98.4)
Level	
Mild	356 (31.1)
Moderate	560 (48.9)
Severe	211 (18.4)
None	18 (1.6)
Restraint behavior	
Score, mean ± SD	1.8 ± 1.6
Prevalence	912 (79.7)
Level	
Mild	608 (53.1)
Moderate	258 (22.5)
Severe	46 (4.0)
None	233 (20.3)
Purging behavior	
Score, mean ± SD	0.6 ± 1.0
Prevalence	506 (44.2)
Level	
Mild	459 (40.1)
Moderate	44 (3.8)
Severe	3 (0.3)
None	639 (55.8)

Cell values represent frequency (%) unless otherwise stated.

The mean ‘restraint’ score was 1.8 ± 1.6, indicating a lower level of restraint compared to ‘concerns’. The ‘restraint’ prevalence was high, as 79.7% of individuals felt some extent of restraint, and distribution of ‘restraint’ levels revealed that a majority of participants exhibited mild (53.1%) or moderate (22.5%) restraint. In contrast, the mean ‘purging’ score was notably lower (0.6 ± 1.0), signifying a relatively lower prevalence and severity of purging behaviors compared to ‘concerns’ and ‘restraint’. Prevalence of ‘purging’ was 44.2%, most respondents felt mild (40.1%) purging behavior, while moderate (3.8%) and severe (0.3%) purging were less prevalent.

### Characteristics Associated with Post-MBS Eating Disorders

Table 7 presents the multiple logistic regression analyses of the associations between patient and surgical characteristics and severe ‘concerns’, severe ‘restraint’ behavior, and moderate to severe ‘purging’ behavior. The odds of severe ‘concerns’ were lower with older age (OR = 0.98, 95% CI 0.96–1.00, *p* = 0.013) and higher pre-operative BMI (OR = 0.96, 95% CI 0.93–0.98, *p* = 0.001). Conversely, severe ‘concerns’ were positively associated with current BMI (OR = 1.19, 95% CI 1.14–1.24, *p* < 0.001) and time since operation (OR = 1.10, 95% CI 1.02–1.18, *p* = 0.011), but not type of MBS procedure. Severe ‘restraint’ behavior was positively associated with pre-operative BMI only (OR = 1.05, 95% CI 1.00–1.09, *p* = 0.023). Moderate to severe ‘purging’ behavior was not associated with any of the variables under examination.

**Table 7:** Patient and surgical characteristics associated with severe ‘concerns’, severe ‘restraint’ behavior, and moderate to severe ‘purging’ behavior

Characteristic	Severe ‘concerns’ about shape/weight/eating			Severe ‘restraint’ behavior			Moderate/severe ‘purging’ behavior		
	OR(SE)	95% CI	*p*	OR(SE)	95% CI	*p*	OR(SE)	95% CI	*p*
(Intercept)	0.02 (0.59)	(0.01–0.05)	< *0.001*	0.00 (1.03)	(0.00–0.03)	< *0.001*	0.01 (1.04)	(0.00–0.06)	< *0.001*
Sex									
Female (reference)									
Male	0.64 (0.25)	(0.38–1.02)	0.067	0.39 (0.54)	(0.12–1.01)	0.084	0.82 (0.43)	(0.33–1.78)	0.637
Age (years)	0.98 (0.01)	(0.96–1.00)	*0.013*	1.02 (0.02)	(0.99–1.05)	0.222	1.02 (0.02)	(0.99–1.05)	0.270
Pre-operative BMI	0.96 (0.01)	(0.93–0.98)	*0.001*	1.05 (0.02)	(1.00–1.09)	*0.023*	1.01(0.02)	(0.96–1.05)	0.743
Current BMI	1.19 (0.02)	(1.14–1.24)	< *0.001*	0.99 (0.03)	(0.93–1.05)	0.782	1.03 (0.03)	(0.97–1.09)	0.351
Time since surgery (years)	1.10 (0.04)	(1.02–1.18)	*0.011*	0.90 (0.08)	(0.77–1.04)	0.181	0.95 (0.07)	(0.82–1.09)	0.448
Procedure									
SG, non-ring-augmented (reference)									
OAGB ^*a*^	0.65 (0.32)	(0.34–1.18)	0.179	0.90 (0.49)	(0.30–2.18)	0.837	1.14 (0.46)	(0.42–2.61)	0.770
rSG	0.17 (1.08)	(0.01–0.94)	0.103	0.98 (1.05)	(0.05–5.10)	0.983	1.04 (1.05)	(0.06–5.31)	0.969
RYGB	0.63 (0.96)	(0.06–3.11)	0.629	3.32 (0.80)	(0.49–13.46)	0.136	1.67 (1.07)	(0.09–9.10)	0.631

Multiple logistic regression analyses. *OR*, odds ratio; *SE*, standard error; *CI*, confidence interval; *BMI*, body mass index; *SG*, sleeve gastrectomy; *OAGB*, one-anastomosis gastric bypass; *rSG*, ringed-augmented sleeve gastrectomy; *RYGB*, Roux-en-Y gastric bypass; ^*a*^ ring-augmented and non-ring-augmented OAGB were grouped together, as ring-augmented variety comprised only a few cases. Italicized cells indicate statistical significance

## Discussion

After MBS, some individuals experience inadequate weight loss or weight recurrence over time [26]. The less-than-adequate responses after MBS may be associated with multiple factors, including new or persistent eating pathology after surgery that could influence WL [9, 26, 65, 66]. The significance of recurrence and new cases of post-MBS ED has been recognized, with the possibility of emergence of new pathological ED in the post-operative period [66–69].

However, despite the high obesity rates and a considerable increase in MBS across MENA, to date, there is no self-reported Arabic questionnaire to appraise post-MBS ED. Although interviews have been the traditional choice [70], the increasing numbers of MBS patients and the expertise required for the interviews render their use impractical [30]. Unsurprisingly, the number of ED that develop after MBS, particularly partial sub-syndrome cases, is probably underreported [71].

In the present study, the translation, cultural adaptation, validity of the constructs, discriminant validity, and internal consistency of the Arabic questionnaire EDABS-Q-Arabic18 were assessed among 1145 post-MBS random patients. To our knowledge, this study is the first to generate an Arabic culturally adapted version of the Eating Disorder Examination-Self-report Questionnaire Bariatric Surgery Version (EDABS-Q) [34] and appraise its psychometric profile. Our main findings were that EDABS-Q-Arabic18 is a culturally appropriate and reliable clinical tool with valid constructs and high discriminant validity for assessing post-MBS among Arabic-speaking patients. Below, we discuss the findings in detail.

In terms of language, in line with others, we translated and validated the EDABS-Q-Arabic18 using Modern Standard Arabic for better comprehension by many Arabic-speaking MBS patients [42], hence overcoming different linguistic characteristics when used in future across MENA.

As for the time of administration, patients completed the Arabic questionnaire on average 3.4 ± 2.1 years after MBS (range:1–12 years), providing sufficient time after surgery where many patients were still losing weight and were still in the ‘honeymoon phase’ after surgery. Such time lag is important, as patients reach a weight plateau and improvements in eating-related attitudes begin to erode [72–74]. This longer time duration after MBS was selected to provide more opportunity for the EDABS-Q-Arabic18 to be able to detect post-MBS ED should they have existed, hence minimizing type II error and generating a rigorous account of the questionnaire’s performance. Short postoperative durations are not conducive to the detection of ED, as MBS seems to promote successful treatment of self-reported ED symptoms during the first post-operative year [75]. Hence, the duration the current study provided is important, as a systematic review of the measurement of post-MBS disordered eating using questionnaires and/or interviews found that the duration of follow-up varied from immediately after surgery to 14.8 years post-surgery [76]; the average length of follow-up was 29.5 months [77]. Our average post-MBS duration before completing the questionnaire was 40.8 months.

Pertaining to the number of factors in the EFA solution, the metrics of 14-, 9-, and 7-factor solutions (Appendix II–IV) overestimated the number of factors. Hence, the 3-factor EFA solution was selected, confirmed by CFA, and congruent with the four subscales of the original Eating Disorder Examination items: dietary restraint, eating concerns, weight concerns, and shape concerns [78].

Regarding validation and psychometric profiles, when developing and validating health, social, and behavioral scales or translated tools, best practice guidelines recommend undertaking EFA and CFA [79]. Despite this, a recent comparison of the interview to questionnaire responses for assessment of post-MBS ED did not conduct both and did not present direct evidence to support the rationale for their questionnaire’s 7-domain structure used among the 30 patients [34]. Likewise, validation of a new tool to quantify patients’ compliance to post-MBS dietary and lifestyle suggestions, the Eating Behavior after Bariatric Surgery Questionnaire, did not appear to have conducted both [58]. In contrast, the post-MBS eating behavior Arabic version questionnaire validated to estimate patients’ adherence to dietary and lifestyle recommendations [42]; and validation of the obstructive sleep apnea‐18 quality of life Arabic version questionnaire both conducted EFA [80]; although only the latter undertook CFA. We conducted both EF and CFA analyses that provided direct evidence and rationale to support the 3-factor 18-item structure that generated three theoretically sound latent factors of the EDABS-Q-Arabic18; and illustrated the potential interrelations between those latent factors in the final structural model. Others seem not to have undertaken such structural model [34].

As for the reliability analysis, the internal consistency, Cronbach’s alpha of the EDABS-Q-Arabic18 was > 0.6, amounting to 0.77 for the ‘concerns’ factor, 0.60 for the ‘restraint’, and 0.63 for the ‘purging’ factor. A comparison of the interview to questionnaire in assessing post-MBS ED did not report Cronbach’s alpha [34], an important statistic in this type of research. For instance, a validation of the obstructive sleep apnea‐18 quality of life questionnaire Arabic version used Cronbach’s alpha and reported the tool’s internal consistency at levels between 0.58 and 0.86 [80] and the post-MBS eating behavior Arabic version questionnaire to estimate patients’ adherence to dietary and lifestyle recommendations after MBS reported the internal consistency Cronbach’s alpha at 0.85 [42].

We analyzed the patient’s demographic and pre-/post-operative anthropometry features, as well as surgical characteristics associated with each factor of the Arabic version EDABS-Q-Arabic18, to provide clues to clinicians to identify patients at risk of post-MBS ED. We are not aware of studies that undertook such analysis. For instance, the authors of the English-published version of the Eating Disorder Examination-Self-report Questionnaire Bariatric Surgery Version (EDABS-Q) [34] did not provide any patient or surgical characteristics of individuals likely to be at risk of ED after MBS. However, other research on the characterization of eating disorders after MBS assessed a case series of 12 individuals [81]. Their cases were all women; female patients comprised most of our series, and hence, we extracted their values for a more valid comparison (Appendix V).

We agree with this study [81], as their patients were aged 23–69 (*M* = 46.8; SD = 16.6), which was within our female sample’s age range of 18–71 years (*M* = 39.6; SD = 9.5); their pre-surgery BMI range was 40.4–79.4 kg/m^2^ (*M* = 50.1; SD = 11.0), again within limits of our findings of pre-surgery BMI range of 23.8–92.6 kg/m^2^ (*M* = 47.3; SD = 7.9), and in support of our observation that the odds of severe ‘concerns’ were lower for those higher pre-operative BMI (OR = 0.94, 95% CI 0.92–0.97, *p* < 0.001). The findings of this study [81] also noted that these patients with ED exhibited ‘marked fear of weight gain’, supporting our EDABS-Q-Arabic18's ‘concerns’ with weight dimension, and their [81] ‘persistent behaviors that interfere with weight gain’ is in agreement with our ‘restraint’ behavior for weight control, both generated by the exploratory factor analysis and confirmed by the confirmatory factor analysis. Furthermore, three of their patients [81] reported engaging in chewing and spitting, concurring with the ‘purging’ behavior for the weight control dimension of the EDABS-Q-Arabic18.

We noted that the prevalence of ‘concerns’ with shape/weight/eating was notably high, where 98.4% of individuals experienced some concerns, albeit the majority were of mild (31.1%) or moderate (48.9%) severity. We used a 7-point ordinal scale (items rated using 0–6 points) as with the original English questionnaire [34], and our mean score was 2.9 ± 1.3. This score compared favorably with findings from Brazil, where very recent research on ED and weight regain in post-MBS patients (also rated using 0–6 points) reported that concerns with shape, weight, and eating across their sample were 4.18 ± 1.58, 3.26 ± 1.51, and 2.17 ± 1.57, respectively [82]. Our scores are far from the maximum score since 2.5 is considered healthy status [75]. Notwithstanding, 18.4% of our series expressed severe concerns with shape, weight, and eating which is of clinical significance.

### Limitations, Strengths, and Future Research

The current study has limitations. It was undertaken at one institution in one Arabic-speaking country among a sample comprising primarily females who had undergone predominately sleeve gastrectomy as a primary procedure. Generalizations to other Arabic-speaking nations should exercise caution. It would have been beneficial to incorporate broader psychological assessments in addition to the demographic and surgical variables that were included. The total variance explained by the three-factor solution was relatively modest, suggesting that additional factors than those explored by the current study could have a role. The study focused on post-operative MBS patients; the inclusion of preoperative data for comparison could add insights. While a comprehensive appraisal of the EDABS-Q-Arabic18's psychometrics was conducted, it did not evaluate convergent validity vis-a-vis a reference standard e.g., the interview, or its responsiveness, the tool’s capacity to identify changes over time or in response to treatment, hence further augmenting its research and clinical utility and comprehensiveness. The cross-sectional design of the current study did not allow for an assessment of changes in EDABS-Q-Arabic18 over time.

The study has many strengths grounded in its rigorous methodological approach. To minimize linguistic variability, modern standard Arabic was used as it is the literary standard across MENA. To minimize operative variability, the same surgeon performed all MBS procedures, eliminating inter-surgeon operative variability that might influence the outcomes and hence ED that might follow. To ensure robustness, the statistical methods adhered to best practices [50, 79], enhancing the reliability and validity of the findings. Employing EFA and CFA on separately randomized data subsets avoided overfitting and robustly validated the questionnaire’s constructs. During the exploratory phase, the use of principal axis factoring and robust maximum likelihood estimation to handle data non-normality, meticulous outlier removal, and exploration of multiple factor solutions using retention methods including Kaiser criterion, scree test, optimal parallel analysis, and minimum average partials ensured robust comprehensive evaluations of potential structures. In the confirmatory phase, the use of the first-order factor model allowed correlations between latent factors to reflect hypothesized construct interrelations. The final structural model and fit indices (*χ*^2^, CFI, TLI, RMSEA, and SRMR) were reported for model adequacy. The discriminant validity of constructs was excellent, confirming the distinctiveness of each factor; and Cronbach’s alpha internal consistency for the items of the final three factors solidified the methodological integrity. Logistic regression revealed patients’ demographic and pre-/post-operative anthropometry and surgical features associated with each factor of the EDABS-Q-Arabic18.

Future research would benefit from appraising the EDABS-Q-Arabic18's performance and its further validation across more diverse Arabic-speaking post-MBS patients with different cultural and socioeconomic contexts, including males and patients who had undergone a greater variety of MBS and/or revisional procedures, whilst incorporating a wide range of psychological assessments and behavioral measures to better control for potential confounding and to clarify these relationships. Test–retest reliability and convergent validity should also be assessed, as well as exploring its responsiveness to temporal changes (pre- to post-operative or over time), with longitudinal studies to comprehend how EDABS-Q-Arabic18 evolves in response to various life events. Findings from such research would enhance its generalizability, robustness, and applicability, thus enriching the understanding of eating behavior dynamics following MBS and across temporal timeframes and the convergence of its findings with other tools to further strengthen the instrument’s psychometric robustness. Moreover, integrating the EDABS-Q-Arabic18 with broader quality-of-life assessments could provide a more holistic evaluation of bariatric surgery outcomes. Future research should also explore the effects of other and additional factors than those explored by the current study, particularly psychological features that could be linked to the outcome, or item refinement to enhance the total variance explained by the current three-factor solution. Very recently, a study of shape discrepancy, weight bias internalization, and ED psychopathology in patients with loss-of-control eating after MBS noted greater discrepancy between current and ideal shape was associated with higher levels of a range of behavioral (ED psychopathology), cognitive (weight bias internalization), and psychological/somatic (depression) concerns [83]. Further investigations are needed to progress the scientific knowledge in this field and to establish effective prevention and control strategies.

## Conclusion

As the number of patients who undertake MBS continues to rise, it is critical that clinicians are vigilant to the possibility of the emergence of a variety of eating pathologies that can develop, particularly since many are frequently not reported due to their sub-syndrome appearance. The Arabic version of the Eating Disorder Examination-Self-report Questionnaire Bariatric Surgery EDABS-Q-Arabic18 is a culturally appropriate and reliable clinical tool comprising valid constructs with high discriminant validity and for assessing eating disorders after MBS among Arabic-speaking patients. It is foreseen that this easy-to-administer and time-saving questionnaire will function as an important tool for identifying eating pathology among this and similar populations and addressing a significant gap in the eating pathology literature among Arabic-speaking post-bariatric patients.

## Supplementary Information

Below is the link to the electronic supplementary material.

ESM 1 (318 KB DOCX)

## Data Availability

The datasets presented in this study can be provided upon reasonable request to the corresponding author and agreement of the institution where the research was implemented.
