# Correction: Translation and Psychometric Evaluation of the Arabic Version of the Eating Disorders After Bariatric Surgery Questionnaire (EDABS‑Q‑Arabic18)

**DOI:** 10.1007/s11695-025-08019-9

**Published:** 2025-06-27

**Authors:** Mohamed Hany, Kareem El-Ansari, Walid El Ansari

**Affiliations:** 1 Alexandria University, Alexandria, Egypt; 2 Madina Women’s Hospital, Alexandria University, Alexandria, Egypt; 3 St. George’s University, St. George’s, Grenada; 4 Ajman University, Ajman, United Arab Emirates

**Correction: Obesity Surgery**

10.1007/s11695-025-07910-9

The authors would like to correct the designation of the corresponding author. Dr. Walid El Ansari and Dr. Mohamed Hany are both co-corresponding authors.

