# Supplementary material for: Translation and Psychometric Evaluation of the Arabic Version of the Eating Disorders After Bariatric Surgery Questionnaire (EDABS‑Q‑Arabic18)
Source: Obes Surg. 2025 Jun 17;35(8):2887–903. doi: 10.1007/s11695-025-07910-9 (PMC12380645; doi:10.1007/s11695-025-07910-9)
Supplement: Supplementary file 1 — (318 KB DOCX) [file 11695_2025_7910_MOESM1_ESM.docx]

**Supplementary Information**

**Appendix I.** Correlation matrix of the 41 questionnaire items
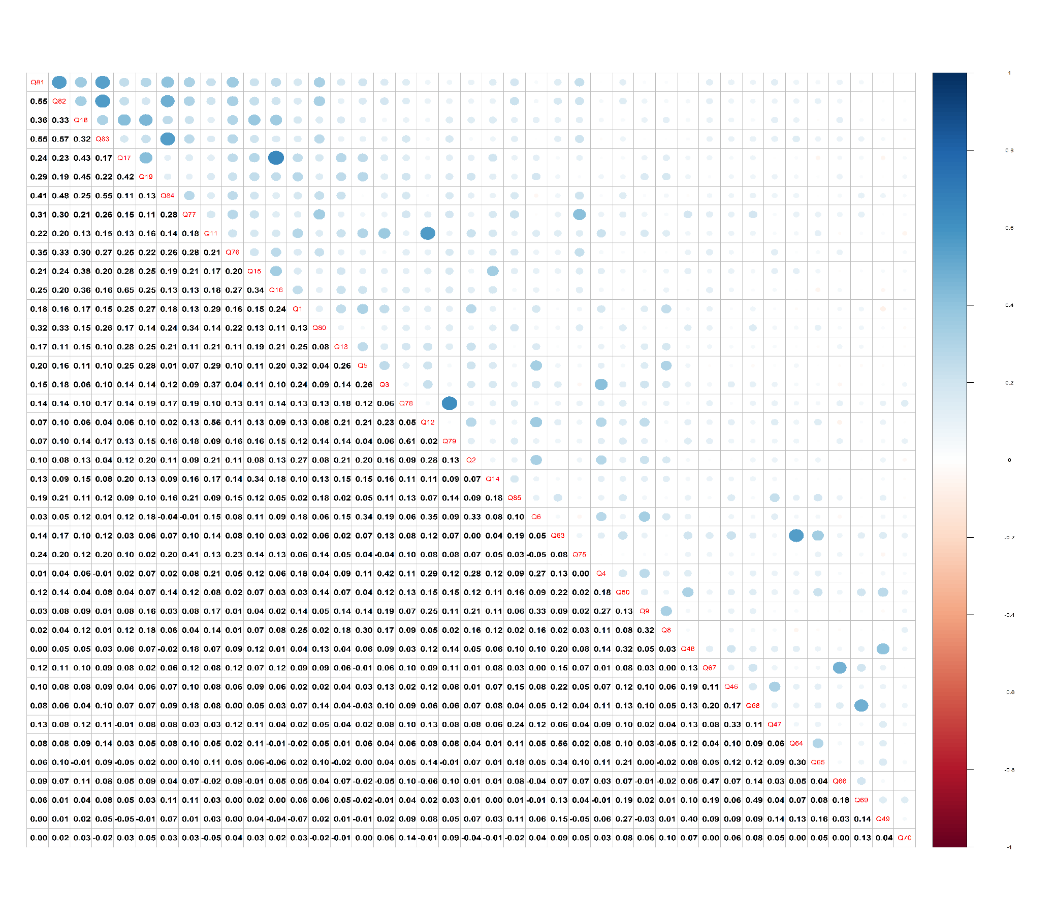


**Appendix II.** Exploratory factor analysis, 14-factor solution

| **Domain** | **Item** | **PA1** | **PA4** | **PA8** | **PA3** | **PA5** | **PA11** | **PA12** | **PA7** | **PA6** | **PA9** | **PA14** | **PA10** | **PA2** | **PA13** | ***h^2^*** |
| --- | --- | --- | --- | --- | --- | --- | --- | --- | --- | --- | --- | --- | --- | --- | --- | --- |
| ‘Concerns’ about |  |  |  |  |  |  |  |  |  |  |  |  |  |  |  |  |
| Shape | Q13 |  |  |  |  |  |  |  |  |  |  |  |  |  |  | 0.22 |
|  | Q15 |  | 0.44 |  |  |  |  |  |  |  |  |  |  |  |  | 0.33 |
|  | Q17 |  | 0.82 |  |  |  |  |  |  |  |  |  |  |  |  | 0.68 |
|  | Q18 |  | 0.49 |  |  |  |  |  |  |  |  |  |  |  |  | 0.50 |
|  | Q79 |  |  |  |  | 0.70 |  |  |  |  |  |  |  |  |  | 0.55 |
|  | Q82 | 0.65 |  |  |  |  |  |  |  |  |  |  |  |  |  | 0.56 |
|  | Q83 | 0.77 |  |  |  |  |  |  |  |  |  |  |  |  |  | 0.61 |
|  | Q84 | 0.71 |  |  |  |  |  |  |  |  |  |  |  |  |  | 0.53 |
| Weight | Q19 |  |  |  |  |  |  |  |  |  |  |  |  |  |  | 0.37 |
|  | Q78 |  |  |  |  | 0.86 |  |  |  |  |  |  |  |  |  | 0.73 |
|  | Q80 |  |  |  |  |  |  |  |  |  |  |  |  |  |  | 0.29 |
|  | Q81 | 0.59 |  |  |  |  |  |  |  |  |  |  |  |  |  | 0.56 |
| Eating | Q14 |  |  |  |  |  |  |  |  |  |  |  |  |  |  | 0.15 |
|  | Q16 |  | 0.71 |  |  |  |  |  |  |  |  |  |  |  |  | 0.52 |
|  | Q75 |  |  |  |  |  | 0.49 |  |  |  |  |  |  |  |  | 0.32 |
|  | Q76 |  |  |  |  |  |  |  |  |  |  |  |  |  |  | 0.29 |
|  | Q77 |  |  |  |  |  | 0.70 |  |  |  |  |  |  |  |  | 0.58 |
| ‘Restraint’ behavior |  |  |  |  |  |  |  |  |  |  |  |  |  |  |  |  |
| For weight control | Q1 |  |  |  |  |  |  |  |  |  |  |  |  |  |  | 0.29 |
|  | Q3 |  |  |  |  |  |  | 0.71 |  |  |  |  |  |  |  | 0.58 |
|  | Q5 |  |  |  |  |  |  |  |  |  |  |  |  |  | 0.48 | 0.51 |
|  | Q8 |  |  |  |  |  |  |  |  |  |  | 0.60 |  |  |  | 0.40 |
|  | Q11 |  |  | 0.62 |  |  |  |  |  |  |  |  |  |  |  | 0.57 |
| To avoid physical discomfort | Q2 |  |  |  |  |  |  |  |  |  |  |  |  |  |  | 0.29 |
|  | Q4 |  |  |  |  |  |  | 0.47 |  |  |  |  |  |  |  | 0.43 |
|  | Q6 |  |  |  |  |  |  |  |  |  |  |  |  | 0.45 |  | 0.41 |
|  | Q9 |  |  |  |  |  |  |  |  |  |  |  |  |  |  | 0.34 |
|  | Q12 |  |  | 0.82 |  |  |  |  |  |  |  |  |  |  |  | 0.70 |
| ‘Purging’ behavior |  |  |  |  |  |  |  |  |  |  |  |  |  |  |  |  |
| For weight control | Q46 |  |  |  |  |  |  |  |  |  |  |  | 0.44 |  |  | 0.28 |
|  | Q48 |  |  |  |  |  |  |  | 0.65 |  |  |  |  |  |  | 0.48 |
|  | Q50 |  |  |  |  |  |  |  |  |  |  |  |  |  |  | 0.28 |
|  | Q63 |  |  |  | 0.86 |  |  |  |  |  |  |  |  |  |  | 0.76 |
|  | Q65 |  |  |  |  |  |  |  |  |  |  |  |  |  |  | 0.25 |
|  | Q66 |  |  |  |  |  |  |  |  |  | 0.75 |  |  |  |  | 0.57 |
|  | Q68 |  |  |  |  |  |  |  |  | 0.69 |  |  |  |  |  | 0.52 |
|  | Q70 |  |  |  |  |  |  |  |  |  |  |  |  |  |  | 0.08 |
|  | Q85 |  |  |  |  |  |  |  |  |  |  |  |  |  |  | 0.23 |
| To avoid physical discomfort | Q47 |  |  |  |  |  |  |  |  |  |  |  | 0.70 |  |  | 0.49 |
|  | Q49 |  |  |  |  |  |  |  | 0.63 |  |  |  |  |  |  | 0.43 |
|  | Q64 |  |  |  | 0.65 |  |  |  |  |  |  |  |  |  |  | 0.43 |
|  | Q67 |  |  |  |  |  |  |  |  |  | 0.62 |  |  |  |  | 0.45 |
|  | Q69 |  |  |  |  |  |  |  |  | 0.69 |  |  |  |  |  | 0.52 |

**Appendix III.** Exploratory factor analysis, 9-factor solution

| **Domain** | **Item** | **PA1** | **PA2** | **PA4** | **PA5** | **PA3** | **PA7** | **PA6** | **PA9** | **PA8** | ***h^2^*** |
| --- | --- | --- | --- | --- | --- | --- | --- | --- | --- | --- | --- |
| ‘Concerns’ about |  |  |  |  |  |  |  |  |  |  |  |
| Shape | Q13 |  |  |  |  |  |  |  |  |  | 0.21 |
|  | Q15 |  |  | 0.42 |  |  |  |  |  |  | 0.28 |
|  | Q17 |  |  | 0.83 |  |  |  |  |  |  | 0.66 |
|  | Q18 |  |  | 0.48 |  |  |  |  |  |  | 0.42 |
|  | Q79 |  |  |  | 0.79 |  |  |  |  |  | 0.63 |
|  | Q82 | 0.73 |  |  |  |  |  |  |  |  | 0.56 |
|  | Q83 | 0.78 |  |  |  |  |  |  |  |  | 0.59 |
|  | Q84 | 0.67 |  |  |  |  |  |  |  |  | 0.45 |
| Weight | Q19 |  |  | 0.41 |  |  |  |  |  |  | 0.37 |
|  | Q78 |  |  |  | 0.76 |  |  |  |  |  | 0.58 |
|  | Q80 |  |  |  |  |  |  |  |  |  | 0.22 |
|  | Q81 | 0.68 |  |  |  |  |  |  |  |  | 0.55 |
| Eating | Q14 |  |  |  |  |  |  |  |  |  | 0.14 |
|  | Q16 |  |  | 0.70 |  |  |  |  |  |  | 0.50 |
|  | Q75 |  |  |  |  |  |  |  |  |  | 0.22 |
|  | Q76 |  |  |  |  |  |  |  |  |  | 0.29 |
|  | Q77 |  |  |  |  |  |  |  |  |  | 0.41 |
| ‘Restraint’ behavior |  |  |  |  |  |  |  |  |  |  |  |
| For weight control | Q1 |  |  |  |  |  |  |  |  |  | 0.30 |
|  | Q3 |  | 0.42 |  |  |  |  |  |  |  | 0.27 |
|  | Q5 |  |  |  |  |  |  |  |  |  | 0.31 |
|  | Q8 |  |  |  |  |  |  |  |  |  | 0.20 |
|  | Q11 |  | 0.69 |  |  |  |  |  |  |  | 0.50 |
| To avoid physical discomfort | Q2 |  |  |  |  |  |  |  |  |  | 0.23 |
|  | Q4 |  | 0.41 |  |  |  |  |  |  |  | 0.27 |
|  | Q6 |  |  |  |  |  |  |  |  |  | 0.33 |
|  | Q9 |  |  |  |  |  |  |  |  |  | 0.23 |
|  | Q12 |  | 0.68 |  |  |  |  |  |  |  | 0.47 |
| ‘Purging’ behavior |  |  |  |  |  |  |  |  |  |  |  |
| For weight control | Q46 |  |  |  |  |  |  |  |  |  | 0.15 |
|  | Q48 |  |  |  |  |  | 0.49 |  |  |  | 0.31 |
|  | Q50 |  |  |  |  |  |  |  |  |  | 0.25 |
|  | Q63 |  |  |  |  | 0.82 |  |  |  |  | 0.70 |
|  | Q65 |  |  |  |  |  |  |  |  |  | 0.23 |
|  | Q66 |  |  |  |  |  |  |  |  | 0.66 | 0.45 |
|  | Q68 |  |  |  |  |  |  | 0.60 |  |  | 0.41 |
|  | Q70 |  |  |  |  |  |  |  |  |  | 0.06 |
|  | Q85 |  |  |  |  |  |  |  |  |  | 0.18 |
| To avoid physical discomfort | Q47 |  |  |  |  |  | 0.40 |  |  |  | 0.18 |
|  | Q49 |  |  |  |  |  | 0.45 |  |  |  | 0.24 |
|  | Q64 |  |  |  |  | 0.68 |  |  |  |  | 0.44 |
|  | Q67 |  |  |  |  |  |  |  |  | 0.68 | 0.51 |
|  | Q69 |  |  |  |  |  |  | 0.69 |  |  | 0.50 |

**Appendix IV.** Exploratory factor analysis, 7-factor solution

| **Domain** | **Item** | **PA1** | **PA2** | **PA5** | **PA3** | **PA4** | **PA6** | **PA7** | ***h^2^*** |
| --- | --- | --- | --- | --- | --- | --- | --- | --- | --- |
| ‘Concerns’ about |  |  |  |  |  |  |  |  |  |
| Shape | Q13 |  |  |  |  |  |  |  | 0.21 |
|  | Q15 |  |  | 0.41 |  |  |  |  | 0.28 |
|  | Q17 |  |  | 0.81 |  |  |  |  | 0.64 |
|  | Q18 |  |  | 0.51 |  |  |  |  | 0.40 |
|  | Q79 |  |  |  |  | 0.81 |  |  | 0.65 |
|  | Q82 | 0.72 |  |  |  |  |  |  | 0.55 |
|  | Q83 | 0.76 |  |  |  |  |  |  | 0.58 |
|  | Q84 | 0.66 |  |  |  |  |  |  | 0.44 |
| Weight | Q19 |  |  | 0.43 |  |  |  |  | 0.35 |
|  | Q78 |  |  |  |  | 0.72 |  |  | 0.53 |
|  | Q80 |  |  |  |  |  |  |  | 0.21 |
|  | Q81 | 0.68 |  |  |  |  |  |  | 0.54 |
| Eating | Q14 |  |  |  |  |  |  |  | 0.14 |
|  | Q16 |  |  | 0.71 |  |  |  |  | 0.50 |
|  | Q75 |  |  |  |  |  |  |  | 0.17 |
|  | Q76 |  |  |  |  |  |  |  | 0.28 |
|  | Q77 |  |  |  |  |  |  |  | 0.34 |
| ‘Restraint’ behavior |  |  |  |  |  |  |  |  |  |
| For weight control | Q1 |  |  |  |  |  |  |  | 0.29 |
|  | Q3 |  | 0.49 |  |  |  |  |  | 0.27 |
|  | Q5 |  | 0.40 |  |  |  |  |  | 0.32 |
|  | Q8 |  |  |  |  |  |  |  | 0.20 |
|  | Q11 |  | 0.57 |  |  |  |  |  | 0.39 |
| To avoid physical discomfort | Q2 |  | 0.44 |  |  |  |  |  | 0.23 |
|  | Q4 |  | 0.50 |  |  |  |  |  | 0.28 |
|  | Q6 |  | 0.50 |  |  |  |  |  | 0.30 |
|  | Q9 |  | 0.43 |  |  |  |  |  | 0.23 |
|  | Q12 |  | 0.65 |  |  |  |  |  | 0.45 |
| ‘Purging’ behavior |  |  |  |  |  |  |  |  |  |
| For weight control | Q46 |  |  |  |  |  |  |  | 0.12 |
|  | Q48 |  |  |  |  |  |  |  | 0.24 |
|  | Q50 |  |  |  |  |  |  |  | 0.21 |
|  | Q63 |  |  |  | 0.73 |  |  |  | 0.56 |
|  | Q65 |  |  |  | 0.41 |  |  |  | 0.23 |
|  | Q66 |  |  |  |  |  |  |  | 0.16 |
|  | Q68 |  |  |  |  |  | 0.60 |  | 0.38 |
|  | Q70 |  |  |  |  |  |  |  | 0.04 |
|  | Q85 |  |  |  |  |  |  |  | 0.16 |
| To avoid physical discomfort | Q47 |  |  |  |  |  |  |  | 0.09 |
|  | Q49 |  |  |  |  |  |  |  | 0.17 |
|  | Q64 |  |  |  | 0.65 |  |  |  | 0.40 |
|  | Q67 |  |  |  |  |  |  |  | 0.20 |
|  | Q69 |  |  |  |  |  | 0.67 |  | 0.44 |

**Appendix V.** Characteristics of female patients of the sample (N=943)

| **Characteristic** | **Value** |
| --- | --- |
| Demographics |  |
| Age (years) | 39.6 ± 9.5 (18-71) |
| Surgery |  |
| Time since MBS (years) | 3.4±2.1 (1-12) |
| Type *^a^* |  |
| Non-ring augmented |  |
| Sleeve gastrectomy | 814 (86.3) |
| One anastomosis gastric bypass | 98 (10.4) |
| Roux-en-Y gastric bypass | 13 (1.4) |
| Ring-augmented |  |
| Sleeve gastrectomy | 15 (1.6) |
| One anastomosis gastric bypass | 3 (0.3) |
| Order *^a^* |  |
| Primary | 930 (98.6) |
| Revision | 13 (1.4) |
| Anthropometry |  |
| Preoperative values |  |
| Height (cm) | 164.3 ± 6.7 (145.0-196.0) |
| Weight (kg) | 127.7 ± 23.0 (64.0-260.0) |
| BMI (kg/m²) | 47.3 ± 7.9 (23.8-92.6) |
| Best anthropometric values |  |
| Nadir weight (kg) | 75.4 ± 13.5 (44.0-145.0) |
| Maximum weight lost (kg) | 52.3 ± 18.0 (7.0-175.0) |
| Nadir BMI (kg/m²) | 27.9 ± 4.9 (15.6-59.6) |
| %EWL | 75.0 ± 14.6 (12.4-133.3) |
| %TWL | 40.3 ± 8.6 (8.2-72.7) |
| Current values (at time of study) |  |
| Weight (kg) | 82.2 ± 15.8 (45.0-180.0) |
| BMI (kg/m²) | 30.5 ± 5.7 (17.1-63.8) |
| %EWL | 65.0 ± 16.7 (0.0-125.9) |
| %TWL | 35.0 ± 9.6 (0.0-66.9) |
| Weight recurrence |  |
| Amount (kg) | 6.8 ± 8.7 (0.0-87.0) |
| % of sample with WR *^a^* | 245 (26.0) |
| % WR from max weight lost *^a^* (kg) | 13.3 ± 15.1 (0.0-100.0) |

Cell values represent mean ± SD (minimum–maximum) unless otherwise indicated; *^a^* Cell values represent frequency (%); *MBS* metabolic and bariatric surgery; *max* maximum; *BMI*, body mass index; *%EWL*, percentage excess weight loss; *%TWL*, percentage total weight loss; *WR*, weight recurrence (≥ 20% weight regain from maximum weight lost)
